# SUPPORT Tools for Evidence-informed Policymaking in health 6: Using research evidence to address how an option will be implemented

**DOI:** 10.1186/1478-4505-7-S1-S6

**Published:** 2009-12-16

**Authors:** Atle Fretheim, Susan Munabi-Babigumira, Andrew D Oxman, John N Lavis, Simon Lewin

**Affiliations:** 1 Norwegian Knowledge Centre for the Health Services, P.O. Box 7004, St. Olavs plass, N-0130 Oslo, Norway; Section for International Health, Institute of General Practice and Community Medicine, Faculty of Medicine, University of Oslo, Norway; 2 Norwegian Knowledge Centre for the Health Services, P.O. Box 7004, St. Olavs plass, N-0130 Oslo, Norway; 3 Centre for Health Economics and Policy Analysis, Department of Clinical Epidemiology and Biostatistics, and Department of Political Science, McMaster University, 1200 Main St. West, HSC-2D3, Hamilton, ON, Canada, L8N 3Z5; 4 Norwegian Knowledge Centre for the Health Services, P.O. Box 7004, St. Olavs plass, N-0130 Oslo, Norway; Health Systems Research Unit, Medical Research Council of South Africa

## Abstract

*This article is part of a series written for people responsible for making decisions about health policies and programmes and for those who support these decision makers*.

After a policy decision has been made, the next key challenge is transforming this stated policy position into practical actions. What strategies, for instance, are available to facilitate effective implementation, and what is known about the effectiveness of such strategies? We suggest five questions that can be considered by policymakers when implementing a health policy or programme. These are: 1. What are the potential barriers to the successful implementation of a new policy? 2. What strategies should be considered in planning the implementation of a new policy in order to facilitate the necessary behavioural changes among healthcare recipients and citizens? 3. What strategies should be considered in planning the implementation of a new policy in order to facilitate the necessary behavioural changes in healthcare professionals? 4. What strategies should be considered in planning the implementation of a new policy in order to facilitate the necessary organisational changes? 5. What strategies should be considered in planning the implementation of a new policy in order to facilitate the necessary systems changes?

## About STP

*This article is part of a series written for people responsible for making decisions about health policies and programmes and for those who support these decision makers. The series is intended to help such people ensure that their decisions are well-informed by the best available research evidence. The SUPPORT tools and the ways in which they can be used are described in more detail in the Introduction to this series *[1]. *A glossary for the entire series is attached to each article *(see Additional File 1). *Links to Spanish, Portuguese, French and Chinese translations of this series can be found on the SUPPORT website *http://www.support-collaboration.org. *Feedback about how to improve the tools in this series is welcome and should be sent to: *STP@nokc.no.

## Scenarios

*Scenario 1: You are a senior civil servant with responsibility for the rollout of a new reform programme in the health services. You want to ensure that implementation takes place as effectively as possible*.

*Scenario 2: You work in the Ministry of Health and have been instructed to prepare an implementation plan for the rollout of the government's recently adopted reform programme for the health services. You wish to explore what types of strategies to consider in such a plan*.

*Scenario 3: You work in an independent unit that supports the Ministry of Health in its use of evidence in policymaking. You are preparing a document on the effects of various interventions that could be included in a national implementation strategy for the new health services reform programme, and need guidance on how to do this*.

## Background

For policymakers (Scenario 1), this article suggests a number of questions that they might ask their staff to consider when the implementation of a new policy is being planned.

For those who support policymakers (Scenarios 2 and 3), this article suggests a number of questions that we believe are worth considering when discussing programme implementation and potentially useful approaches.

The process of translating policy into practice can be challenging and is often done in an unsystematic way. Careful planning is needed to prevent otherwise good policies being hampered by poor implementation. But the implementation process is not always a straightforward one: it may involve a complex set of actions at various levels of the health system as well as within communities.

Two key issues should be considered by those responsible for policy implementation, namely: "How can the activities related to the policy option be implemented to produce real changes on the ground?", and "Which strategies are available to facilitate effective implementation?"

A number of entry points can be used when planning policy implementation. Our suggested approach entails first identifying barriers to implementation, and then tailoring the implementation strategies to address the barriers - and facilitators - that are found.

**Figure 1:**
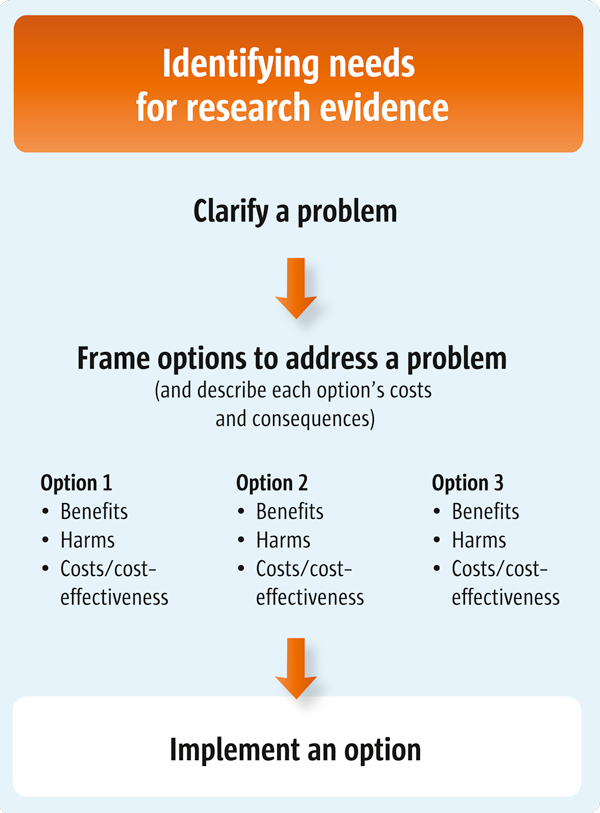
**Clarifying evidence needs**.

This article is the third of three articles about clarifying evidence needs (see also Articles 4 and 5). (Figure 1 outlines the processes involved in clarifying these needs).

## Questions to consider

1. What are the potential barriers to the successful implementation of a new policy?

2. What strategies should be considered in planning the implementation of a new policy in order to facilitate the necessary behavioural changes among healthcare recipients and citizens?

3. What strategies should be considered in planning the implementation of a new policy in order to facilitate the necessary behavioural changes in healthcare professionals?

4. What strategies should be considered in planning the implementation of a new policy in order to facilitate the necessary organisational changes?

5. What strategies should be considered in planning the implementation of a new policy in order to facilitate the necessary systems changes?

### 1. What are the potential barriers to the successful implementation of a new policy?

A useful starting point for anyone wanting to elicit change is the identification of likely barriers to change. Knowing what - and where - the major hurdles are that may affect successful implementation is useful during the planning process. These challenges will often vary from policy to policy, and between different contexts. Both research findings on barriers to policy implementation in other settings, and lessons learnt from previous experiences may be informative, but they may not be sufficient.

There is no standard approach to identifying barriers to change. This process is often done informally by taking *perceived *barriers into account and in an implicit and unsystematic way. We propose a more structured approach to identifying barriers.

The people who will be affected by a policy - the stakeholders - are the ones likely to be best placed to foresee possible barriers to policy implementation. A number of methods can be used to explore the views of stakeholder groups about new policies including, for example, a 'mixed methods approach' for undertaking a so-called 'diagnostic analysis'. This approach may include brainstorming, focus group discussions, interviews and other qualitative methods, or a combination of these. Such activities can provide new insights into stakeholders' perceptions and identify both barriers - and facilitators - to policy implementation. Surveys can also be useful. For example, respondents could be asked to rate the extent to which a list of potential barriers actually represents obstacles to change. Practical examples of such processes are provided in Table 1[2,3].

**Table 1:** Examples of how barriers to policy implementation can be identified

**Accessing antiretroviral therapy (ART) in Tanzania **[3]
ART has been freely available in selected reference hospitals in Tanzania since 2005 as part of the national government's policy to make ART more widely accessible. Making medicines available, however, does not automatically result in patients being able to access them. In order to identify barriers to ART access in a particular setting where the drugs were made available, a team of researchers conducted focus group discussions with community members and in-depth interviews with treatment seekers. The researchers found that "transportation and supplementary food costs, the referral hospital's reputation for being unfriendly and confusing, and difficulties in sustaining long-term treatment would limit accessibility." They noted too that a "fear of stigma framed all [patient] concerns, posing challenges for contacting referrals those who did not want their status disclosed or who had expressed reluctance to identify a "treatment buddy" as required by the programme".
**Cholesterol-screening in the United States **[2]
American researchers examined the barriers to participation in cholesterol screenings in both adults and children in West Virginia in the United States. Using the theory of 'planned behaviour' as a conceptual framework to provide a model for understanding decision making within particular belief systems and cultures, the researchers postulated that an individual's intention to perform an action is a central factor in determining whether an individual *will *perform that action. The researchers conducted semi-structured interviews using interview guides designed to elicit information relevant to the key constructs of the theory of planned behaviour. Their findings suggest that environmental, financial, *and *attitudinal barriers affected levels of participation in these health screenings. These include concerns about the outcomes of testing, the use of needles, privacy, a lack of knowledge in the community, and local traditional cultural beliefs.

Several frameworks and checklists have been developed to help identify potential barriers to implementing health interventions. These are often based on a combination of behavioural theories, empirical data, and common sense, and may be useful tools in guiding the process of identifying barriers. Some frameworks cover a broad range of potential barriers in various parts of the health system. For example, in one framework, barriers are categorised according to the level at which the constraints operate [4]. These levels include: the household and community, delivery of health services, health sector policy and strategic management, public policies cutting across sectors, and environmental and contextual characteristics. Examples of barriers identified at each of these levels are shown in Table 2.

**Table 2:** Constraints to improving access to priority health interventions, by level (from [4])

Level of constraint	Types of constraint
I. Community and household level	• Lack of demand for effective interventions
	• Barriers to the use of effective interventions (physical, financial, social)

II. Health services delivery level	• Shortage and distribution of appropriately qualified staff
	• Weak technical guidance, programme management and supervision
	• Inadequate drugs and medical supplies
	• Lack of equipment and infrastructure, including poor accessibility of health services

III. Health sector policy and strategic management level	• Weak and overly-centralised systems for planning and management
	• Weak drug policies and supply system
	• Inadequate regulation of pharmaceutical and private sectors and improper industry practices
	• Lack of inter-sectoral action and partnership for health between government and civil society
	• Weak incentives to use inputs efficiently and respond to user needs and preferences
	• Reliance on donor funding that reduces flexibility and ownership
	• Donor practices that damage country policies

IV. Public policies cutting across sectors	• Government bureaucracy (civil service rules and remuneration, centralised management systems, civil service reforms)
	• Poor availability of communication and transport infrastructure

V. Environmental and contextual characteristics	• Governance and overall policy framework
	- Corruption, weak government, weak rule of law and
	- enforceability of contracts
	- Political instability and insecurity
	- Low priority attached to social sectors
	- Weak structures for public accountability
	- Lack of free press
	• Physical environment
	- Climatic and geographic predisposition to disease
	- Physical environment unfavourable to service delivery

We have adopted a similar approach by focusing on constraints to policy implementation at three levels in the health system:

• Among healthcare recipients and citizens

• Among healthcare professionals

• At the organisational level

Once the likely barriers to policy implementation have been identified, the next step is to identify implementation strategies or interventions that can address these (Table 3 shows examples of possible links between barriers and interventions among healthcare recipients and citizens). The choice of strategies should also be guided by the available evidence of their effectiveness and costs, as well as stakeholders' views, etc. The issue of how to find and assess evidence that may be relevant is addressed in other articles in this series [5-9].

**Table 3:** Examples of possible links between barriers and interventions among healthcare recipients and citizens

Identified barrier to policy implementation	Possible interventions to address identified barriers
Current programmes are ineffective or of uncertain effectiveness	• Review the components of ongoing programmes, as well as the evidence from systematic reviews regarding other possible options for evidence of effectiveness • Conduct rigorous evaluations of programmes

Poor satisfaction with care	• Improve evidence-based strategies to improve the quality of care delivered

The relevant services are not within physical reach of some patients/citizens in need of them	• Creation of new services • Hiring of new personnel • Redistribution of resources

Denial of the severity of their problem	• Education and community awareness programmes

Transportation costs	• Provision of transportation or financial support for transport

### 2. What strategies should be considered in planning the implementation of a new policy in order to facilitate the necessary behavioural changes among healthcare recipients and citizens?

The behaviour of healthcare recipients and citizens, particularly in relation to the use of health services (e.g. under-utilisation, non-adherence to recommended lifestyle changes or treatment schedules, etc.), may be a potentially significant obstacle to successful policy implementation. It is necessary to understand why the targeted recipients behave in particular ways as this will influence the choices they make in utilising health services. Well-conducted qualitative studies can provide insights into the behaviour of healthcare recipients [10].

One framework that can be used to identify factors that may influence the behaviour of healthcare recipients and citizens was proposed by a WHO working group on adherence to long-term therapies. They suggested five dimensions to consider [11]:

• Socio-economic related factors

• Health system and healthcare-related factors

• Therapy-related factors

• Factors-related to the particular health conditions of patients

• Patient-related factors

As these factors are related more specifically to clinical interventions, they may be particularly useful when considering barriers to the delivery of care arrangements. For example, some of the socio-economic factors that can affect adherence to treatment among patients with tuberculosis include: a lack of effective social support networks and unstable living circumstances, cultural and lay beliefs, ethnicity, gender, age, the high cost of medication and transport, and the role of criminal justice [11,12].

The Cochrane Consumer and Communication Review Group has extensively documented the effects of interventions to improve interactions between consumers and healthcare providers and systems, and has developed a taxonomy of interventions that target healthcare recipients and citizens [13]. This may be helpful when conceptualising and considering what kinds of interventions to use. The taxonomy includes:

• Provision of education or information

• Support for changing behaviour

• Support for developing skills and competencies

• Personal support

• Facilitation of communication and decision making, and

• System participation

Several studies and reviews have evaluated the effects of interventions that address constraints to effective health service delivery at the level of healthcare recipients and citizens. In one review, the authors found positive effects from community participation in overcoming such constraints [14]. In this instance, community participation was obtained using a variety of intervention approaches, including: health education (e.g. meetings, group teachings), encouraging a participative approach (mobilising leaders and stakeholders to understand and buy into the intervention), using an outreach strategy (targeting households and high-risk groups), and the training and supervision of providers (e.g. nurses and/or mothers). These interventions resulted in increased health-related knowledge and community empowerment and improved coverage in immunisation and sanitation practices.

Financial incentives, such as conditional cash transfers, may be worth considering if socio-economic related barriers are seen as playing an important role. This is because evidence, particularly from low- and middle-income countries, indicates that these may have an impact on the use of health services [15] (see Table 4 for details). A further illustrative example of evidence on the impacts of financial incentives is provided in Table 5[16].

**Table 4:** Summary of key findings from systematic review of conditional cash transfer programmes in low- and middle-income countries [15]

• Overall, the evidence suggests that conditional cash transfer (CCT) programmes are effective in increasing the use of preventive services for children and women, and sometimes in improving health status
• Only one study evaluated the effect of providing different amounts of cash (from $1 to $3). The overall effect of the increase was a near doubling in the proportion of people returning for their HIV-test results (72% of people who had received incentives compared to 39% of those who had not)
• While the flows of money required for CCT programmes may be significant, the actual transfer budget may account for between only 4 to 28% of a total programme budget
• The cost-effectiveness of CCT programmes compared with classic supply-side interventions (e.g. improving the quantity and quality of infrastructure and services) has not been examined, as most CCT programmes have been implemented in settings with relatively adequate (health) infrastructures
• Unanticipated perverse effects can occur. For instance, one programme reported unexpected increases in the fertility rate when CCTs were used, possibly because only pregnant women were eligible for the subsidy

**Table 5:** Example of evidence that can inform the design of an implementation strategy targeted at healthcare recipients and citizens

**Cash rewards for learning HIV-status, in Malawi **[16]
Potential barriers to obtaining results from HIV-testing include the monetary costs of time and travel, and psychological costs (for example, stress, worry and fear, or the experience of social stigma). Monetary incentives may compensate directly for time and transport costs - and potentially for any psychological costs incurred. In a field experiment in rural Malawi, individuals were randomly assigned monetary incentives to learn their HIV results after testing. Where no incentives were offered, one-third of those tested obtained their results. In contrast, where small monetary incentives were provided, two-thirds went to obtain their HIV-test results.

If patient-related factors, such as a lack of information appear to be important barriers to policy implementation, interventions to improve information provision might be worth considering. A systematic review has shown that mass media interventions, for example, "can encourage increased utilisation of health services". But this finding should be approached with caution given that the study was based almost exclusively on studies from high-income countries [17] and therefore may not be applicable to other settings.

### 3. What strategies should be considered in planning the implementation of a new policy in order to facilitate the necessary behavioural changes in healthcare professionals?

The implementation of a policy or programme will often require changes in the behaviour of those healthcare professionals responsible for implementing the policy on the ground. Changes in professional behaviour do not always necessarily happen automatically. Active and directed approaches may therefore be necessary. The identification of barriers to change or factors that influence professional practice may help to inform the design of interventions for policy implementation. Cabana and colleagues conducted a systematic review of research addressing barriers to guideline adherence among physicians [18] and identified seven main categories of barriers. These can be used as a framework for identifying barriers to policy implementation among healthcare professionals:

• Lack of awareness

• Lack of familiarity

• Lack of agreement

• Lack of self-efficacy

• Lack of outcome-expectancy

• Inertia of previous practice

• External barriers

Examples of how identifying barriers can inform implementation are provided in Table 6.

**Table 6:** Examples of possible links between barriers and interventions among healthcare professionals

Identified barrier to policy implementation	Possible interventions to address identified barriers
Lack of knowledge	• Information delivery methods (educational outreach, training)

Disagreement with policy	• Identify opinion leaders who can act as advocates for the new policy

Time consuming	• Offer economic compensation

The Effective Practice and Organisation of Care (EPOC) Review Group in the Cochrane Collaboration has developed a taxonomy of provider-targeted interventions which provides an overview of the types of interventions that may be considered for implementation purposes [19]. These are:

• Educational materials

• Educational meetings

• Educational outreach visits

• Local opinion leaders

• Local consensus processes

• Peer review

• Audit and feedback

• Reminders and prompts

• Tailored interventions

• Patient-mediated interventions

• Multi-faceted interventions

Several strategies aimed at achieving behavioural change among healthcare professionals have been rigorously assessed [20-23]. Typically, these have taken the form of evaluations of guideline implementation strategies targeted directly at healthcare professionals. Most, but not all, have been conducted in high-income settings [24]. The findings demonstrate that many interventions can influence professional behaviour effectively to a modest or moderate extent. But passive interventions, such as the circulation of guidelines or the hosting of educational meetings, seem only to have smaller impacts. Educational outreach visits and multi-faceted interventions that specifically target identified barriers to change are among the more promising approaches.

Financial incentives may be used as a means of influencing professional behaviour but these have been evaluated almost entirely in high-income settings. These can be effective in influencing individual healthcare professionals when simple and well-defined behavioural goals are provided, such as increases in the delivery of immunisations - at least in the short term [25]. However, several potentially negative consequences of such programmes have been identified and the use of financial incentives may not necessarily be cost-effective.

Regulatory measures are inexpensive and potentially effective means of eliciting changes in professional behaviour but may be poorly received by professional groups [26]. The impact of regulations per se as a means of achieving behavioural change among healthcare professionals has not been reviewed systematically, therefore available knowledge about their effectiveness is limited [27].

See Table 7 for further illustrative examples of evidence on the effects of interventions to achieve behavioural change among healthcare professionals [28,29].

**Table 7:** Examples of evidence that can inform the design of implementation strategies targeted at healthcare professionals

**Financial incentives to health workers to increase institutional deliveries in India **[28]
In 2005, the Indian government introduced the Janani Suraksha Yojana (JSY) programme which aimed to reduce maternal and neonatal mortality through the promotion of institutional deliveries. Cash payments to community health workers (ASHAs) for institutional deliveries among women under their care was one of the key programme components. Since the introduction of the programme, many Indian states have seen a substantial increase in institutional deliveries. However, an evaluation of one such programme suggests that the financial incentives for ASHA probably played a small if any role in this.
**Educational outreach visits to improve asthma care in South Africa **[29]
South African researchers found that two 30-minute educational outreach visits to general practitioners conducted by a trained pharmacist led to clinically important improvements in symptom scores for children with asthma.

### 4. What strategies should be considered in planning the implementation of a new policy in order to facilitate the necessary organisational changes?

Many organisational change strategies see the measures that should be taken as steps in a process that leads to change. Defining why there is a need for change and identifying barriers to change are tasks that are typically included in this process.

Pexton has proposed a list of the most common barriers to organisational change and this can also be used as a framework for barrier-identification [30]:

• Cultural complacency (resistance or scepticism)

• Lack of communication

• Lack of alignment and accountability

• Passive or absent leadership

• Micro-management

• An overloaded workforce

• Inadequate systems and structures

Ways to address each of these types of barriers are suggested in Table 8.

**Table 8:** Proposed list of common organisational barriers to change (adapted from [30])

Barriers	Strategies to address barriers
Cultural complacency (resistance or scepticism)	• Deliver a few quick 'measurable wins' to demonstrate why change is needed

Lack of communication	• Develop a communication strategy targeted to identified communication barriers in the organisation

Lack of alignment and accountability	• Institute appropriate management structures

Passive or absent leadership	• Engage leaders in the proposed changes

Micro-management	• Empower the team and establish vision for the organisation among team members

Overloaded workforce	• Demonstrate the benefits of rethinking workflow to team members and of using new processes or technologies to reduce non value-added steps

Inadequate systems and structures	• Institute appropriate systems and structures to support the initiative

Lack of control plans to measure and sustain results	• Develop mechanisms to assess progress and maintain any positive results attained

Examples of the tools and approaches often recommended to organisations assessing preparations for change include [31]:

• Analytic models for understanding complexity, interdependence and fragmentation (such as Weisbord's six-box organisational model, the 7S model, and process models)

• Tools for assessing why change is needed, such as SWOT analysis

• Tools for determining who and what can change, such as force field analysis and total quality management

• Tools for making changes, such as organisational development, action research and project management

Most commonly used organisational change strategies are based almost entirely on theory, or else on one-off applications and opinion. Sometimes these are supplemented with case studies or anecdotes, mainly from high-income settings [31]. Evidence about the effectiveness of these strategies is hard to come by, making it difficult to predict whether or not a specific method is likely to lead to the desired organisational change.

Although the impacts of such change management strategies are uncertain, they may still be useful as processes allowing for active reflection on how change in an organisation can be facilitated.

### 5. What strategies should be considered in planning the implementation of a new policy in order to facilitate the necessary systems changes?

When a policy is to be implemented, changes at the general level of a health system may be necessary. These may include changes to governance arrangements, financial arrangements and delivery arrangements [32]. For example, when considering the financing of a policy option, should all costs be incurred by the government, or are additional sources of funding needed? Can the current system cope with the additional bureaucratic or logistical workload, or is a new mechanism needed to deliver the service? The body of evidence on how to implement such changes is small: those making decisions will usually have to draw on case studies and experiences in other jurisdictions. For particular policy implementation issues systematic reviews may be useful, such as those related to the costs of scaling up interventions [33] or factors that may affect the sustainability of health programmes [34]. In a recent overview, the authors summarised the evidence from systematic reviews on the effects of governance, financial and delivery arrangements, and implementation strategies that have the potential to improve the delivery of cost-effective interventions in primary health care in LMICs [27].

When identifying the need for system changes it may be useful to review the components of a health system and to identify where changes are required. Table 9 shows a framework that can be used as a starting point for such analyses [35].

**Table 9:** Various components of health systems (adapted from Lavis et al [35])

Delivery arrangements	Financial arrangements	Governance arrangements
• To whom care is provided and the efforts made to reach them (such as interventions to ensure culturally appropriate care)	• Financing - e.g. how revenue is raised for programmes and services (such as through community-based insurance schemes)	• Policy authority - who makes policy decisions (such as whether such decisions are centralised or decentralised)?

• By whom care is provided (such as providers working autonomously versus those who work as part of multidisciplinary teams)	• Funding - e.g. how clinics are paid for the programmes and services they provide (such as through global budgets)	• Organisational authority - e.g. who owns and manages clinics (such as whether private for-profit clinics exist)

• Where care is provided - e.g. whether care is delivered in the home or community health facilities	• Remuneration - e.g. how providers are remunerated (such as via capitation)	• Commercial authority - e.g. who can sell and dispense drugs and how they are regulated

• With what information and communication technology is care provided - e.g. whether record systems are conducive to providing continuity of care	• Financial incentives - e.g. whether patients are paid to adhere to care plans	• Professional authority - e.g. who is licensed to deliver services, how their scope of practice is determined, and how they are accredited

• How the quality and safety of care is monitored - e.g. whether quality-monitoring systems are in place	• Resource allocation - e.g. whether drug formularies are used to decide which medications patients receive for free	• Consumer and stakeholder involvement - who is invited to participate in policymaking processes from outside government and how their views are taken into consideration

## Conclusion

A consideration of the aspects of policy implementation described in this article should enable policymakers and those who support them to employ a structured approach that includes the use of research findings in the design of implementation strategies. Currently, implementation plans often are developed on an ad hoc basis, and are rarely informed by available evidence. As the approach outlined in this article is not widely used, we encourage the sharing of experiences in this area of evidence-informed policy implementation.

## Resources

### Useful documents and further reading

Shared decision-making in health care. Achieving evidence-based patient choice (2^nd ^edition, Edited by Edwards A and Elwyn G). Oxford University Press, 2009.

Changing Professional Practice (Edited by: Thorsen T and Mäkelä M) Copenhagen: Danish Institute for Health Services Research and Development, 1999. http://www.dsi.dk/projects/cpp/Monograph/DSI9905.pdf

Grol R, Wensing M, Eccles M. Improving Patient Care: The Implementation of Change in Clinical Practice. Oxford: Elsevier, 2005.

Fretheim A, Schünemann HJ, Oxman AD. Improving the use of research evidence in guideline development: 15. Disseminating and implementing guidelines. Health Research Policy and Systems 2006, 4:27. http://www.health-policy-systems.com/content/4/1/27

NorthStar - how to design and evaluate healthcare quality improvement interventions. The ReBEQI Collaboration 2005: http://www.rebeqi.org/?pageID=34&ItemID=35

Iles V, Sutherland K. Organisational Change. A review for health care managers, professionals and researchers. 2001. London, National Co-ordinating Centre for NHS Service Delivery and Organisation R & D http://www.sdo.nihr.ac.uk/files/adhoc/change-management-review.pdf.

### Links to websites

Cochrane Consumers and Communication Review Group Resource Bank: http://www.latrobe.edu.au/chcp/cochrane/resourcebank/index.html - The Cochrane Consumers and Communication Review Group is part of the Cochrane Collaboration, an international, non-profit organisation that aims to help people make well-informed decisions about healthcare. The Consumers and Communication Review Group co-ordinates the production of systematic reviews of interventions which affect consumers' interactions with healthcare professionals, services and researchers. This resource bank is a list of Cochrane systematic reviews relevant to people's health communication and participation needs, and has been produced by manually searching The Cochrane Library.

Cochrane Effective Practice and Organisation of Care (EPOC) Review Group: http://www.epoc.cochrane.org/en/index.html - EPOC is a Collaborative Review Group of the Cochrane Collaboration and produces systematic reviews of educational, behavioural, financial, regulatory and organisational interventions that are designed to improve healthcare professional practice and the organisation of health care services.

## Competing interests

The authors declare that they have no competing interests.

## Authors' contributions

AF prepared the first draft of this article. SMB, ADO, JNL and SL contributed to drafting and revising it.

## Supplementary Material

Additional file 1 Glossary Click here for file
